# Accuracy of Guided Surgery and Real-Time Navigation in Temporomandibular Joint Replacement Surgery

**DOI:** 10.3390/dj9080087

**Published:** 2021-08-02

**Authors:** Michael-Tobias Neuhaus, Alexander-Nicolai Zeller, Alexander K. Bartella, Anna K. Sander, Bernd Lethaus, Rüdiger M. Zimmerer

**Affiliations:** 1 Department of Oral and Maxillofacial Surgery, Leipzig University, Liebigstraße 12, 04103 Leipzig, Germany; alexander.bartella@medizin.uni-leipzig.de (A.K.B.); anna.sander@medizin.uni-leipzig.de (A.K.S.); bernd.lethaus@medizin.uni-leipzig.de (B.L.); ruediger.zimmerer@medizin.uni-leipzig.de (R.M.Z.); 2 Department of Oral and Maxillofacial Surgery, Hannover Medical School, Carl-Neuberg-Str. 1, 30625 Hannover, Germany; zeller.alexander@mh-hannover.de

**Keywords:** temporomandibular joint disorders, total joint replacement, virtual surgical planning, guided surgery, real-time navigation, 3D-printing

## Abstract

Background: Sophisticated guided surgery has not been implemented into total joint replacement-surgery (TJR) of the temporomandibular joint (TMJ) so far. Design and in-house manufacturing of a new advanced drilling guide with vector and length control for a typical TJR fossa component are described in this in vitro study, and its accuracy/utilization was evaluated and compared with those of intraoperative real-time navigation and already available standard drilling guides. Methods: Skull base segmentations of five CT-datasets from different patients were used to design drilling guides with vector and length control according to virtual surgical planning (VSP) for the TJR of the TMJ. Stereolithographic models of the skull bases were printed three times for each case. Three groups were formed to compare our newly designed advanced drilling guide with a standard drilling guide and drill-tracking by real-time navigation. The deviation of screw head position, screw length and vector in the lateral skull base have been evaluated (n = 72). Results: There was no difference in the screw head position between all three groups. The deviation of vector and length was significantly lower with the use of the advanced drilling guide compared with standard guide and navigation. However, no benefit in terms of accuracy on the lateral skull base by the use of real-time navigation could be observed. Conclusion: Since guided surgery is standard in implant dentistry and other CMF reconstructions, this new approach can be introduced into clinical practice soon, in order to increase accuracy and patient safety.

## 1. Introduction

Alloplastic replacement (total joint replacement, TJR) has become a standard procedure for the treatment of temporomandibular joint (TMJ) disorders when non-invasive therapy is not feasible or has failed [1,2]. In the early period of TMJ reconstructive surgery, autologous reconstruction was preferred, as other methods were not previously available [3,4,5]. Nowadays, an autograft, which has a success rate of 50% [6], is only feasible for pediatric surgery [7]. The alloplastic reconstruction of an affected TMJ is superior in terms of predictability and stability of reconstruction [8].

Unlike in orthopedics, where alloplastic TJR has been well established for decades, the TJR of the TMJ has suffered from several shortcomings and setbacks during development. This was mainly due to the fact that early TJR systems led to severe and unpredictable complications, and thus failed to serve their purpose [9,10]. By the end of their development, prostheses consisting of mandibular components made of cobalt-chrome or titanium and fossae made of ultrahigh molecular weight polyethylene prevailed. Methods including partial joint replacements have not been able to provide reliable results. Several different TJR systems are currently available. They range from very conventional designs and materials to some that may appear exotic; however, there are standardized off-the-shelf prostheses and patient-matched solutions. These highly individual solutions are planned by computer-aided design (CAD) and produced by computer-aided manufacturing procedures (CAM) such as milling and three-dimensional (3D) printing. The introduction of virtual surgical planning (VSP) for TMJ replacement allows for complex reconstructions, as nearly every shape of implant design is possible [11]. Cases with severe deformities or simultaneous microvascular bony reconstructions can especially benefit from the possibilities of CAD/CAM.

Osseointegration is crucial for the success of all alloplastic implants, dental implants as well as orthopedic implants [12]. Achieving long-term stability in TJR is challenging, though, as most patients with indication for a TJR of the TMJ suffer from distorted anatomy of the jaw and skull base [7]. Patient-matched prostheses can partially overcome these difficulties by a one-fit-only design for specific anatomic situations [13]. This facilitates the avoidance of micromovement-induced connective tissue between bone and implant, with following implant failure. Primary stability of TMJ prostheses always relies on sufficient osteosynthesis. However, in surgical sites with large defects after ablative surgery, trauma, ankylosis or congenital deformities, the possibilities of bony anchorage are restricted, particularly in areas adjacent to multiple sensitive structures, such as the skull base, which makes osteosynthesis highly sophisticated. Mal-angulation of screws at the lateral skull base can lead to intracranial screw positioning and damage to the inner ear. That is why individual cutting and drilling guides were developed, to translate digital planning into the operation theater. Unfortunately, these are only able to identify screw head positions, and none provide any information about the drilling/screw length or vector. As TJR prostheses do not feature locking screws, reusable drilling sleeves like in conventional osteosynthesis are not applicable either. Moreover, in implant dentistry, vector and length control are of the utmost importance. That is why “guided surgery” has been widely used for years [14,15,16].

To improve the intraoperative implementation of VSP in TJR replacement surgery, surgical guides could be endowed with drilling sleeves or cylinders that reflect different vectors, lengths and, if required, stops. In addition, real-time navigation with infrared-tracked drills and instruments for vector and length control could be used as formerly described [17,18]. However, intraoperative real-time navigation provided guidance during dissection and resection at the lateral skull base but less so during drilling [13]. Furthermore, navigation systems always come with certain aberrances arising from their technique limitations [19].

Therefore, this study aimed to develop and assess a new sophisticated type of drilling guide with vector and length control to overcome these inherent issues. Its in-house design and manufacturing are described in detail. The accuracy and predictability of drilling in TMJ replacement surgery on the skull base using this surgical guide and real-time navigation was evaluated.

## 2. Materials and Methods

### 2.1. Virtual Surgical Planning (VSP)

For this study, computed tomography (CT) datasets of five patients were segmented using the planning platform iPlan© CMF (craniomaxillofacial) (iPlan© CMF, Version 3.05, Brainlab, Munich, Germany). Special attention was paid to the skull base and temporal region. These areas were segmented by a combined atlas and threshold-based segmentation method. The surgery models of the five patient cases have been printed three times each. 

**Figure 1:**
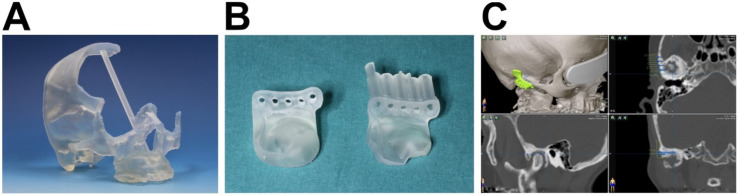
Preparation of model surgery (**A**) A 3D-printed (Clear resin, Form2^®^, Formlabs Inc., Somerville, MA, USA) model of a patient’s skull base with right glenoid fossa and parts of the calvaria for fixation of a navigation reference array (see also Figure 2C). (**B**) The simple (left) and newly designed advanced (right) drilling guides for vector and length control (Clear resin, Form2^®^, Formlabs Inc., Somerville, MA, USA). (**C**) “Preoperative” virtual surgical plan with stereolitographic model of the advanced drilling guide (green-yellow) and trajectory planning of screw positions and vectors (blue lines with dots on each end) using Brainlab^®^ iPlan CMF 3.05.

For the simulation of VSP, favorable positions and vectors for drilling holes were identified according to the standard procedure during VSP in the clinical routine of the TJR of the TMJ in iPlan© CMF. They were marked with the “brush” tool to make them exportable. The generated STL files (stereolitography) of the vectors and temporomandibular joint region were then exported for stereolithographic 3D printing and design of the drilling guide. Three-dimensional printing was conducted in the clinic’s own laboratory using a Form2^®^ printer (Formlabs Inc., Somerville, MA, USA) with Formlabs^®^ Clear resin consisting of acrylic esters (Figure 1A). Supporting structures for 3D printing were generated in PreForm^®^ (Formlabs Inc., Somerville, MA, USA) according to the manufacturer’s specifications. The drilling guides for a typical fossa component were designed in Geomagic^®^ Freeform^®^ (Version 2019, 3D Systems, Rockhill, SC, USA). For CAD, Geomagic^®^ Freeform^®^ STL files were imported and converted into volumetric data. Consequently, a construct similar to a typical fossa component was placed into its designated position. For each case, two guides were designed and printed as described above, one with information for the screw head position only (simple guide) and the other with additional information for vector and length control (advanced guide) (Figure 1B). As the drills’ working length was 24 mm, the preplanned vectors were elongated to 22 mm for the advanced guide. Planned drilling depth was then subtracted from the cylinders. Their center was used to create 22 mm long cylinders with diameters of 2.2 mm and 5.5 mm around them. By Boolean operations, the segmented fossa was subtracted from the fossa component, and 2.2 mm were subtracted from the 5.5 mm cylinder, creating a tube. Differences between the cylinder height and drill length thus lead to the correct drilling depth. After subtracting the 5.5 mm cylinder from the fossa component, the pre-described tube was added to the fossa component. At the tube bases, 2 mm diameter holes were added as an opening for rinsing, to prevent overheating during drilling.

**Figure 2:**
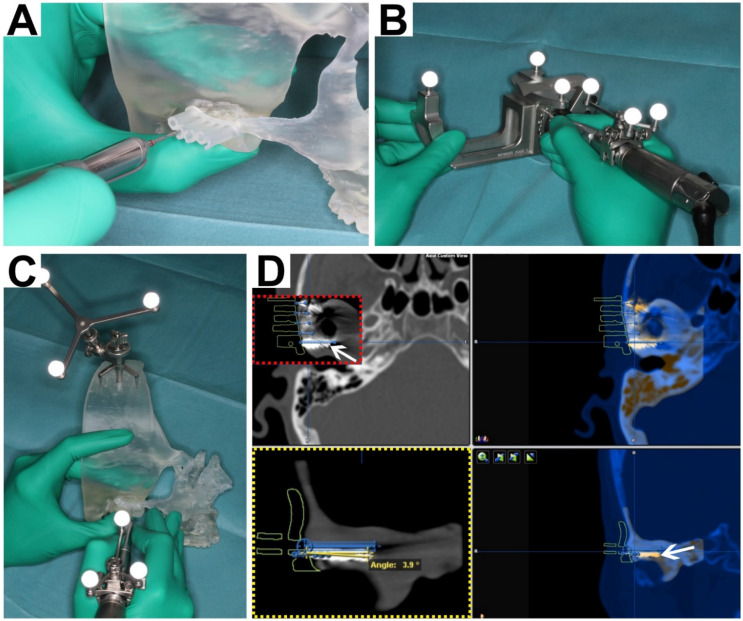
Performing model surgery with and without navigation. (**A**) Guided surgery with advanced drilling guide (group 1) for vector and length control in a patient model of the right TMJ. (**B**) Referencing and setup of navigational instrument/drill-tracking (Brainlab^®^ Kick, Brainlab, Munich, Germany). (**C**) Navigation-tracked drilling for vector and length control using a simple drilling guide. (**D**) Superimposition of preoperative computed tomography dataset, containing the virtual surgical planning, with the postoperative cone beam computed tomography scan of the surgery model. Drill holes were filled with radiopaque root canal sealer (white arrows). Red Box: Planned screw vectors (blue lines with dots on each end). The screw head position [mm], drilling length [mm] and vector [degree] (yellow box) were measured (yellow lines) for deviation from the virtual surgical plan (blue vector).

Using iPlan© CMF, VSP was performed with determination of the aspired screw length, position and vector (Figure 2C), as it would be routinely performed in clinical routine prior to TMJ surgery. The feasibility and VSP of real-time navigation in TMJ replacement surgery have been formerly described [20]. For real-time navigation, holes (1 mm × 1 mm × 1 mm) were placed with registration landmarks on the upper incisor midpoint, the anterior nasal spine, the mesial buccal cusp of the first molars of the upper jaw and the zygomatic process of the temporal bone. Consequently, cone beam CT (CBCT; PaX-Zenith3D, VATECH, Hwaseong, Korea) was conducted and imported into iPlan© CMF. The virtually planned drilling guides of a fictional-patient-matched TMJ prosthesis were imported as well to visualize the planned position (Figure 1C). 

### 2.2. Drilling Procedure

The experimental drilling of the stereolithographic-printed models for fixation of the fossa component of a TMJ prosthesis was performed by a single experienced maxillofacial surgeon. A surgical drill with 1.8-mm diameter and 24-mm length was used in a Microspeed Uni micromotor and Microspeed Uni micro 150 handpiece (Aesculap^®^, B. Braun, Melsungen, Germany). The handpiece functioned as a length stop at 22 mm. 

In each group, drilling was performed for each of the five patient cases, which leads to a total of 72 drill holes (four cases with five drill holes each and one case with four drill holes). In group 1 (advanced guide), drilling guides with length and vector control were used (Figure 2A). In group 2 (simple guide), drilling guides with screw head position were used to determine the drilling hole position. The drilling vector and length were assessed by the performing surgeon in reference to VSP. In group 3 (navigation), drilling was performed using the guide of group 2 and Brainlab^®^ Kick (Brainlab, Munich, Germany) optical intraoperative real-time navigation system for vector and length control (Figure 2B,C). For navigation setup, the above mentioned bony facial landmarks were approached and the handpiece was registered for drill-tracking. Navigation accuracies <1 mm were accepted.

### 2.3. Data Evaluation

Drill holes were filled with a radiopaque dental root canal sealer (AH Plus^®^, Dentsply Sirona Deutschland GmbH, Bensheim, Germany). The stereolithographic models were again scanned by CBCT. DICOM datasets of these scans were fused with the formerly created VSP in iPlan© CMF. This allowed for the measurement of the deviation in vector (degree), length (mm) and screw head position (mm) between the planned screw location and the actual drill hole (Figure 2D). Data were collected in Excel^®^ 2016 (Microsoft Corporation, Redmond, WA 98052-6399, USA) and statistical analyses were performed using IBM SPSS (SPSS Statistics 25, IBM, Armonk, NY, USA). Means, medians and standard deviations were calculated. Statistical significance was verified with a Student’s *T*-test at a significance level of 0.05.

## 3. Results

**Figure 3:**
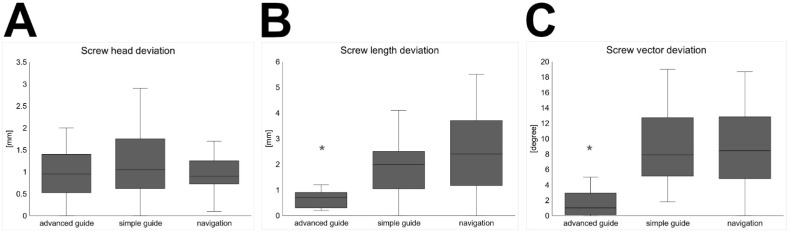
Boxplots of the measured deviation from virtual surgical planning (median, box height: interquartile range, whiskers: lowest and highest value). (**A**) Screw head deviation [mm]. (**B**) Screw length deviation [mm], significant difference of advanced guide to both other groups * = *p* < 0.003. (**C**) Screw vector deviation [degree], significant difference of advanced guide to both other groups * = *p* < 0.001.

In each group, the drilling procedure was performed on five different patient models, resulting in a total of 72 holes. The deviation of the screw head position was measured three-dimensionally. Using the novel advanced guide in group 1 deviation was 0.97 ± 0.58 mm, while using the conventional guide in group 2 deviation it was 1.17 ± 0.58 mm, and using the navigation in group 3 the deviation was 0.95 ± 0.38 mm. No significant difference between all three groups was observed in the position of the screw head (Figure 3A).

The drill hole length differed by 0.66 ± 0.32 mm from the VSP in group 1, 2.15 ± 1.43 mm in group 2 and 2.40 ± 1.55 in group 3. The deviation of the drill hole length was significantly lower in group 1 (advanced guide) than in groups 2 (simple guide) and 3 (navigation) (*p* < 0.003) (Figure 3B). No difference was found between groups 2 and 3. 

A similar pattern of deviation was observed for screw vector deviation, which was significantly lower in group 1 (*p* < 0.001), with 1.9 ± 2.18°. Mean deviation was 8.65 ± 4.78° in group 2 and 8.84 ± 4.74° in group 3 (Figure 3C).

## 4. Discussion

The maximum chewing force loaded to a TMJ prosthesis can reach >400 N/mm² [21]. The primary stability of TJR components is granted by the osteosynthesis used and accounts for the primary success of TMJ implantation. The precise positioning and fixation of TMJ prostheses during implantation have been shown to be essential for long-term surgical success by reducing micromovements and enhancing osseointegration [12]. The use of CAS in TMJ replacement surgery has been proven feasible and was thoroughly described in the literature [22,23,24]. Within this study, we described a new approach using improved surgical guides for vector and length control, which was proven to be superior to standard guide and real-time navigation in terms of accuracy and transformation of VSP. However, no difference between all three groups was observed with regard to screw head deviation. The simple guide, used in groups 2 and 3, seemed to define the screw head position sufficiently. This in vitro study verified previous research, demonstrating that drilling accuracy at the lateral skull base does not benefit from navigation-tracked drilling during surgery [13]. 

As the base area of both types of guides used in this study is identical, no extension of incision during surgery in clinical use should be necessary. On the contrary, the drilling sleeves can prevent soft tissue damage. Water cooling is essential during bone drilling, independently of the use of surgical guides. Nevertheless, there should be more attention on water rinsing when using guides or drilling sleeves. Therefore, holes at the base of drilling sleeves on the advanced guide were added, to facilitate water feeding, which is standard in other drilling guides in CMF surgery [25]. 

This study, though, is limited by its in vitro design. The presented guide has to prove its practicability and value in clinical use in the future. However, since guided surgery has facilitated dental and craniomaxillofacial surgery for years, this improved surgical guide for TMJ replacement surgery should be easily introduced in clinical practice in the near future. However, this in vitro examination is necessary to convince prosthesis manufacturers of its benefits and implementation. It may significantly increase accuracy and patient safety during the TJR of the TMJ with an in-house design and manufacturing at low cost. Contrary to our findings, Gomez et al. [26] have found that intraoperative real-time navigation aids drilling and the insertion of a TJR’s fossa component. Considering that only screw head positions were measured, which were not influenced by navigation, their statement that real-time navigation aids drilling at the lateral skull base can be put into a different context by the results of this study. 

## 5. Conclusions

The presented advanced guide significantly increases surgical precision during screw placement at the lateral skull base with a mean length deviation of 0.66 ± 0.32 mm and a mean vector deviation of 1.9 ± 2.18 degree between VSP and the actually achieved position. This was significantly lower than in control groups. However, navigational drill-tracking was less beneficial than expected. For further improvement of surgical procedures and increased patient safety, this type of advanced guide should be introduced in clinical routine by prosthesis manufacturers. 

## Data Availability

The data presented in this study are available on request from the corresponding author. The data are not publicly available due to data privacy and property rights.

## References

[1] Guarda-Nardini L., Manfredini D., Ferronato G. (2008). Temporomandibular joint total replacement prosthesis: Current knowledge and considerations for the future. Int. J. Oral Maxillofac. Surg..

[2] Aagaard E., Thygesen T. (2014). A prospective, single-centre study on patient outcomes following temporomandibular joint replacement using a custom-made Biomet TMJ prosthesis. Int. J. Oral Maxillofac. Surg..

[3] Smith A.E., Robinson M. (1952). A new surgical procedure in bilateral reconstruction of condyles, utilizing iliac bone grafts and creation of new joints by means of non-electrolytic metal. A preliminary report. Plast. Reconstr. Surg..

[4] Ware W.H., Taylor R.C. (1966). Cartilaginous growth centers transplanted to replace mandibular condyles in monkeys. J. Oral Surg..

[5] Matukas V.J., Szymela V.F., Schmidt J.F. (1980). Surgical treatment of bony ankylosis in a child using a composite cartilage-bone iliac crest graft. J. Oral Surg..

[6] Wolford L.M., Cottrell D.A., Henry C. (1994). Sternoclavicular grafts for temporomandibular joint reconstruction. J. Oral Maxillofac. Surg..

[7] Mercuri L.G. (2006). Total joint reconstruction—Autologous or alloplastic. Oral Maxillofac. Surg. Clin. N. Am..

[8] Henry C.H., Wolford L.M. (1993). Treatment outcomes for temporomandibular joint reconstruction after Proplast-Teflon implant failure. J. Oral Maxillofac. Surg..

[9] Lindqvist C., Söderholm A.L., Hallikainen D., Sjövall L. (1992). Erosion and heterotopic bone formation after alloplastic temporomandibular joint reconstruction. J. Oral Maxillofac. Surg..

[10] Westermark A., Koppel D., Leiggener C. (2006). Condylar replacement alone is not sufficient for prosthetic reconstruction of the temporomandibular joint. Int. J. Oral Maxillofac. Surg..

[11] Elledge R., Mercuri L.G., Speculand B. (2018). Extended total temporomandibular joint replacements: A classification system. Br. J. Oral Maxillofac. Surg..

[12] Sidebottom A.J., Gruber E. (2013). One-year prospective outcome analysis and complications following total replacement of the temporomandibular joint with the TMJ Concepts system. Br. J. Oral Maxillofac. Surg..

[13] Neuhaus M.T., Zeller A.N., Jehn P., Lethaus B., Gellrich N.C., Zimmerer R.M. (2021). Intraoperative real-time navigation and intraoperative three-dimensional imaging for patient-specific total temporomandibular joint replacement. Int. J. Oral Maxillofac. Surg..

[14] Verstreken K., Van Cleynenbreugel J., Marchal G., Van Steenberghe D., Suetens P. (1996). Computer-assisted planning of oral implant surgery: An approach using virtual reality. Stud. Health Technol. Inform..

[15] Jacobs R., Adriansens A., Verstreken K., Suetens P., Van Steenberghe D. (1999). Predictability of a three-dimensional planning system for oral implant surgery. Dentomaxillofac. Radiol..

[16] Flügge T.V., Nelson K., Schmelzeisen R., Metzger M.C. (2013). Three-dimensional plotting and printing of an implant drilling guide: Simplifying guided implant surgery. J. Oral Maxillofac. Surg..

[17] Schmelzeisen R., Gellrich N.C., Schramm A., Schn R., Otten J.E. (2002). Navigation-guided resection of temporomandibular joint ankylosis promotes safety in skull base surgery. J. Oral Maxillofac. Surg..

[18] Schramm A., Gellrich N.C., Schmelzeisen R. (2007). Navigational Surgery of the Facial Skeleton.

[19] Heiland M., Habermann C.R., Schmelzle R. (2004). Indications and limitations of intraoperative navigation in maxillofacial surgery. J. Oral Maxillofac. Surg..

[20] Boccalatte L.A., Nassif M.G., Figari M., Gómez N.L., Argibay M.C., Mancino A.V., Ritacco L.E. (2020). Computer-assisted surgery for replacement of the temporomandibular joint with customized prostheses: Can we validate the results?. Oral Maxillofac. Surg..

[21] Ackland D.C., Robinson D., Redhead M., Lee P.V.S., Moskaljuk A., Dimitroulis G. (2017). A personalized 3D-printed prosthetic joint replacement for the human temporomandibular joint: From implant design to implantation. J. Mech. Behav. Biomed. Mater..

[22] Movahed R., Teschke M., Wolford L.M. (2013). Protocol for concomitant temporomandibular joint custom-fitted total joint reconstruction and orthognathic surgery utilizing computer-assisted surgical simulation. J. Oral Maxillofac. Surg..

[23] Movahed R., Wolford L.M. (2015). Protocol for concomitant temporomandibular joint custom-fitted total joint reconstruction and orthognathic surgery using computer-assisted surgical simulation. Oral Maxillofac. Surg. Clin. N. Am..

[24] Wolford L.M. (2016). Computer-assisted surgical simulation for concomitant temporomandibular joint custom-fitted total joint reconstruction and orthognathic surgery. Atlas Oral Maxillofac. Surg. Clin. N. Am..

[25] Wilde F., Hanken H., Probst F., Schramm A., Heiland M., Cornelius C.-P. (2015). Multicenter study on the use of patient-specific CAD/CAM reconstruction plates for mandibular reconstruction. Int. J. CARS.

[26] Gomez N.L., Boccalatte L.A., Ruiz Á.L., Nassif M.G., Figari M.F., Ritacco L. (2020). Total temporomandibular joint replacement and simultaneous orthognathic surgery using computer-assisted surgery. J. Maxillofac. Oral Surg..

